# The “Intermediate” CD14 + CD16 + monocyte subpopulation plays a role in IVIG responsiveness of children with Kawasaki disease

**DOI:** 10.1186/s12969-021-00573-7

**Published:** 2021-05-31

**Authors:** Yi Seul Kim, Hyun Jin Yang, Seung-Jung Kee, Insu Choi, Kisoo Ha, Katrina K Ki, In Seok Jeong, Hwa Jin Cho

**Affiliations:** 1 Department of Pediatric, Chonnam National University Children’s Hospital, 42 Jaebong ro, Gwangju, South Korea; 2 Department of Pediatrics, Ewha Womans University College of Medicine, Seoul, South Korea; 3 Chonnam National University Medical School, 42 Jaebong ro, Gwangju, South Korea; 4 Department of Laboratory Medicine, Chonnam National University Hwasun Hospital, Hwasun, South Korea; 5 Department of Pediatrics, Korea University Guro Hospital, Korea University College of Medicine, Seoul, Korea; 6 Critical Care Research Group,, The Prince Charles Hospital, Queensland Chermside, Australia; 7 Faculty of Medicine, The University of Queensland, St. Lucia, Queensland Australia; 8 Deparment of Cardiothoracic Surgery, Chonnam National University Hospital and Medical School, 42 Jaebong ro, Gwangju, South Korea

**Keywords:** Kawasaki disease, Immunity, Intermediate monocyte, IVIG responsiveness, IVIG-resistant

## Abstract

**Background:**

Kawasaki disease (KD) is an acute, self-limited febrile illness of unknown cause. Intravenous immunoglobulin (IVIG)-resistance are related to greater risk for permanent cardiac complications. We aimed to determine the correlation between monocytes and the phenotype of KD in relation to IVIG responsiveness in children.

**Materials and methods:**

The study cohort included 62 patients who were diagnosed with KD, 20 non febrile healthy controls (NFC), and 15 other febrile controls (OFC). In all enrolled patients, blood was taken at least 4 times and laboratory tests were performed. In addition, subtypes of monocytes were characterized via flow cytometry.

**Results:**

The numbers of intermediate monocytes were significantly lower in IVIG-resistant group compared to IVIG-responsive group before IVIG infusion (*p* < 0.0001). After infusion, intermediate monocytes decreased in the responsive group, while a trend of increase was observed in the resistant group. Only intermediate monocytes were significant in logistic regression with adjusted OR of 0.001 and *p* value of 0.03.

**Conclusions:**

CD14 + CD16 + intermediate monocyte may play an important role in IVIG responsiveness among KD children. Low starting levels of intermediate monocytes, followed by a dramatic increase post-IVIG infusion during acute phase of KD are associated with IVIG-resistance. Functional studies on intermediate monocyte may help to reveal the pathophysiology.

## Introduction

Kawasaki disease (KD) is an acute, self-limited febrile illness. It is the most common cause of acquired heart disease in children in developed countries, but the causes of this illness are still unknown [1].

If undiagnosed or untreated, permanent cardiac complication such as coronary arterial dilatation (CAL) can occur. The duration of fever and timing of the first treatment is known to contribute to permanent cardiac complications in KD patients. The probability of acquiring this permanent cardiac complication increases if the patient is resistant to intravenous Immunoglobulin (IVIG), which is approximately 10–20 % of the KD population [2, 3].

Although the inflammatory cascade during the acute phase of KD has been extensively studied and associated with monocyte/macrophage activation [4–9] the immune characteristics of IVIG-resistant patients remain to be elucidated regarding IVIG responsiveness. In addition, the role in subtypes of monocytes are limited.

The aim of this study was to determine if there is a correlation between monocytes and the phenotype of KD regarding the responsiveness to IVIG through characterization of KD children immune profiles.

## Materials and methods

### Study population

The study cohort included patients who were diagnosed with KD and required hospital admission. We excluded patients who were transferred for the treatment of refractory KD after the first treatment of KD. Non febrile, healthy controls (NFC) and other febrile controls (OFC) were also included. NFC were those who visited the hospital for routine blood exams including: (i) no history of acute fever within 2 months, (ii) those who came for routine checkups such as antibody check of hepatitis B or anemia check-ups, (iii) prematurity (> 35 weeks). OFC were those who came to the emergency department or outpatient clinic for evaluation of acute febrile illness, such as pneumonia, urinary tract infections, meningitis, and otitis media. All subjects were enrolled at Chonnam National University Children’s Hospital, a tertiary, university-based hospital, from May 1, 2017 to Apr. 30, 2019. The study protocol was approved by the institutional Review Board (CNUH-2017-257) and written informed consent was obtained from all guardians in accordance with the Declaration of Helsinki.

KD was defined with the American Heart Association criteria [3] All hospitalized patients were treated with 2 g/kg of IVIG of single infusion, and with 30–50 mg/kg aspirin during the acute phase which was lowered to 3–5 mg/kg/day 2–3 days after the patients were afebrile. We defined IVIG-resistant patients as those who had persistent fever 36 h after completion of the first IVIG infusion. For patients who are IVIG-resistant, 2nd IVIG of the same dosage was infused. If fever persists 36 h after completion of 2nd IVIG infusion, intravenous methylprednisolone pulse therapy (30 mg/kg/dose) was performed for 3 consecutive days. If fever persists after 3 days of methylprednisolone infusion, 5 mg/kg infliximab was infused.

In all enrolled patients, demographic data including age at diagnosis, sex, body weight, symptomatic phenotype, infused drug, and date of each treatment were recorded. All enrolled patients underwent echocardiography at least 4 times to identify any KD-related cardiac complications.

### Laboratory blood tests and flow cytometric analysis of monocyte subtypes

Blood was taken at each stage of the disease course for all KD patients, and laboratory tests were performed including complete blood counts (CBC), C-reactive protein (CRP), erythrocyte sedimentation rate (ESR). Flow cytometric analysis of monocyte subtypes was also carried out for both KD patients and controls. The cells were stained with BV421-conjugated anti-CD14, PE-conjugated anti-CD16, all from Becton Dickinson Biosciences; San Diego, CA, USA. The subtypes of monocytes were then classified into the classical (CD14 + CD16-), the intermediate (CD14 + CD16+), and the nonclassical (CD14 + CD16++) monocytes.

### Statistical analysis

Continuous variables are expressed as means ± standard deviations. The independent t-test (for normally distributed data) and the Mann–Whitney test (for non-normally distributed data) were used to compare continuous variables between the groups. ANOVA (for normally distributed data) and Kruskal-Wallis test (for non-normally distributed data) were used to compare continuous variables among three groups. For post-hoc analyses, Tukey method was used. In all analyses, p-values < 0.05 were considered to be statistically significant. All analyses were performed using MedCalc Statistical Software ver. 19.1 (MedCalc Software bvba; Ostend, Belgium; http://www.medcalc.org; 2019).

## Results

A total of 222 KD patients were diagnosed and admitted to the hospital during the study period. Among them, 62 patients were enrolled and compared to NFC and OFC.

### Clinical characteristics regarding IVIG responsiveness

The demographics and treatment methods are described in Table 1. Amongst the 62 KD patients, 50 patients were IVIG responsive and 12 patients were IVIG-resistant. Rash (90.3 %) and conjunctivitis (90.3 %) were the most commonly seen manifestations in enrolled KD patients followed by red lip/red tongue (88.7 %), Edema of extremities (66.1 %), desquamation (58.3 %) and cervical lymphadenopathy (54.8 %). However, no statistical difference between IVIG responsive and resistant groups was observed. The total incidence of coronary artery dilatation was 9.7 %, and it was significantly higher in IVIG-resistant group (*n* = 3, 25 %) compared to IVIG responsive group (*n* = 3, 6 %).

**Table 1:** Clinical information of children with Kawasaki disease regarding IVIG responsiveness at admission day

	Total (*n* = 62)	IVIG-responsive (*n* = 50)	IVIG-resistant (*n* = 12)	*P*-Value
Age	2.6 ± 2.2	2.6 ± 2.2	2.5 ± 2.3	0.82
Male (n, %)	35 (56.4 %)	28(56 %)	7(58.3 %)	0.88
Body weight (kg)	14.9 ± 6.3	14.7 ± 5.5	15.6 ± 9.2	0.82
**Symptoms (n, %)**				
Rash	56 (90.3 %)	44(88.0 %)	12(100.0 %)	0.21
Red lip, Red tongue	55 (88.7 %)	43(86.0 %)	12(100.0 %)	0.17
Desquamation	35 (58.3 %)	26(54.2 %)	9(75.0 %)	0.19
Conjunctivitis	56 (90.3 %)	44(88.0 %)	12(100.0 %)	0.21
Cervical lymphadenopathy	34 (54.8 %)	28(56.0 %)	6(50.0 %)	0.71
Edema of extremity	41 (66.1 %)	31(62.0 %)	10(83.3 %)	0.16
Coronary artery dilatation	6 (9.7 %)	3 (25 %)	3 (6 %)	0.04
**Laboratory findings**				
WBC (k/mm3)	15.2 ± 5.8	14.8 ± 6.0	17.5 ± 4.5	0.28
Neutrophil (k/mm3)	10.7 ± 7.5	9.7 ± 5.5	15.5 ± 12.3	0.01
Lymphocyte (k/mm3)	3.6 ± 2.3	3.6 ± 2.3	3.3 ± 2.2	0.63
Monocyte (k/mm3)	0.9 ± 0.5	0.9 ± 0.5	0.8 ± 0.6	0.64
NLR	4.9 ± 4.8	4.2 ± 3.7	7.9 ± 7.4	0.01
Hemoglobin	11.3 ± 1.04	11.3 ± 0.9	11.2 ± 1.5	0.81
Total bilirubin	0.7 ± 0.8	0.5 ± 0.4	1.3 ± 1.4	0.26
Platelet	352.5 ± 113.5	348.3 ± 106.6	370.0 ± 142.8	0.78
Albumin	3.7 ± 0.5	3.7 ± 0.5	3.3 ± 0.4	< 0.01
ESR	71.9 ± 29.7	74.7 ± 30.1	63.7 ± 28.7	0.34
CRP	8.5 ± 5.7	8.0 ± 5.5	10.3 ± 6.3	0.21
**Treatment**				
1st IVIG	60 (96.8 %)	48(96.0 %)	12(100 %)	< 0.01
2nd IVIG	12 (19.3 %)	0(0.0 %)	12(100 %)	< 0.01
Steroid	5 (8.1 %)	0(0.0 %)	5(41.7 %)	< 0.01
Infliximab	1 (1.6 %)	0(0.0 %)	1(8.3 %)	0.04
Aspirin	61 (98.4 %)	49(98.0 %)	12(100.0 %)	0.50
Clopidogrel	2 (3.2 %)	2(4.0 %)	0(0.0 %)	0.50

*CRP* C-reactive protein, *ESR* erythrocyte sedimentation rate, *IVIG* intravenous immunoglobulin, *NLR* neutrophil to lymphocyte ratio, *WBC* white blood cells

### Laboratory findings regarding IVIG responsiveness

Laboratory findings of the 62 patients are summarized in Table 1, comparing between IVIG-responsive and -resistant groups at diagnosis. The absolute counts for total white blood cells (WBC), lymphocytes, and monocytes were not significantly different between groups. The neutrophil counts and the neutrophil to lymphocyte ratio (NLR) were significantly higher in the IVIG-resistant group than IVIG-responsive group (*p* = 0.01 and *p* = 0.01, respectively). The level of albumin was significantly lower (3.3 ± 0.4 g/dL) in IVIG-resistant group compared to IVIG-responsive group (3.7 ± 0.5 g/dL, *P* = 0.001). There was no difference in the levels of hemoglobin, total bilirubin, platelet, ESR, and CRP between the groups.

### Monocytes in children with Kawasaki disease

#### Comparison of monocyte subtype proportions between KD and controls

**Fig. 1:**
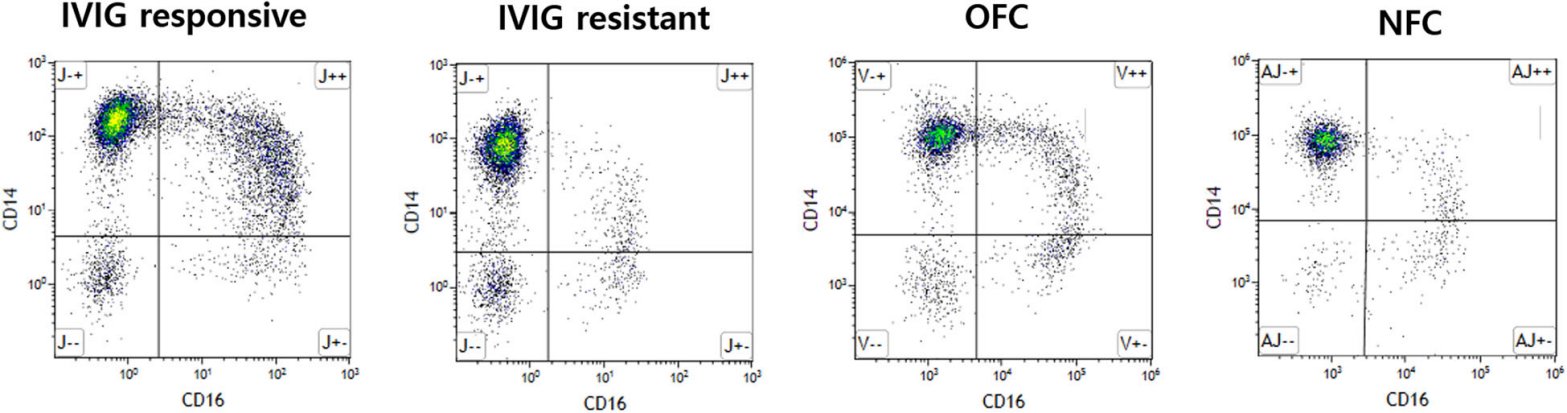
Flow cytometric analyses of monocytes. **a** IVIG-responsive Kawasaki disease, **b** IVIG-resistant Kawasaki disease, **c** Other febrile controls and **d** Non febrile healthy controls

The proportion of monocyte subtypes was compared between KD patients and controls. (Fig. 1; Table 2) The classical monocytes were significantly higher in KD patients and OFC compared to NFC. A lower level of intermediate monocytes was also observed in NFC, while OFC reported the highest level followed by KD patients. No differences were observed for nonclassical monocytes between all groups.

**Table 2:** Monocytes in children with Kawasaki disease and controls

Monocytes	KD (*n* = 62)	NFC (*n* = 20)	OFC (*n* = 15)	*p*-value
Classical (%)	3.6 ± 1.9^a^	1.7 ± 0.7^a^	3.4 ± 1.7^a^	< 0.001
Intermediate (%)	0.7 ± 0.6^ab^	0.3 ± 0.3^a^	1.1 ± 0.7^ab^	< 0.001
Non-classical (%)	0.3 ± 0.3	0.2 ± 0.1	0.2 ± 0.1	0.483

*KD* Kawasaki disease, *NFC* non febrile control, *OFC* other febrile control

^a^, NFC vs. KD and/or OFC, ^b^, KD vs. OFC

#### Comparison of monocyte proportions in children with Kawasaki disease regarding IVIG responsiveness at diagnosis, before IVIG

As summarized in Table 3, only CD14 + CD16 + intermediate monocytes were significantly different between IVIG-responsive and IVIG-resistant groups. Before IVIG infusion, the levels of CD14 + CD16 + monocytes in IVIG-responsive group (0.8 ± 0.6) was significantly higher than IVIG-resistant group (0.3 ± 0.1, *p* < 0.001).

**Table 3:** Monocytes in children with Kawasaki disease regarding IVIG responsiveness

Monocytes	IVIG-responsive(*n* = 50)	IVIG- resistant(*n* = 12)	*p*-value
Classical (%)	3.7 ± 1.8	3.0 ± 2.6	0.26
Intermediate (%)	0.8 ± 0.6	0.3 ± 0.1	< 0.001
Non-classical (%)	0.3 ± 0.4	0.2 ± 0.2	0.12

*IVIG* Intravenous immunoglobulin

### Changes of intermediate monocytes: from acute phase (before (D0) and after IVIG infusion (D2)) to convalescent phase (D56)

**Fig. 2:**
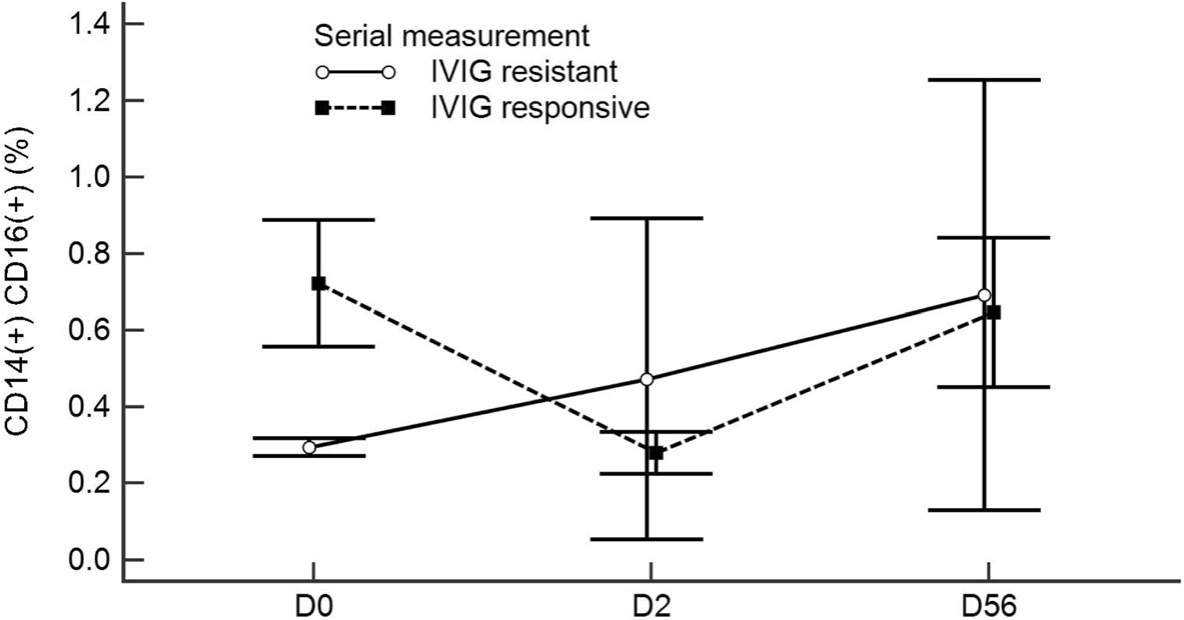
Serial measurement of CD14 + CD16 + intermediate monocytes in IVIG- responsive and IVIG-resistant groups. The time of measurements were D0 (acute phase, at diagnosis, before IVIG infusion), D2 (acute phase, after resolution of fever, after IVIG infusion) and D56 (convalescent phase, 2 months after diagnosis). * <0.01

The changes of CD14 + CD16 + intermediate monocytes during acute phase (before and after IVIG) and convalescent phase in relation to IVIG responsiveness are described in Fig. 2. The percentage of CD14 + CD16 + intermediate monocytes decreased after IVIG infusion in the responsive group, while in the resistant group no significant changes were observed. The intermediate monocytes in both responsive and resistant groups increased to similar levels during the convalescent phase (after 2 months of disease onset).

### Logistic regression and ROC curve at admission day regarding IVIG responsiveness

Only intermediate monocytes were significant in logistic regression with adjusted OR *f* < 0.01 and *p* value of 0.02 (Table 4).

**Table 4:** Logistic regression of variables at admission day between IVIG- responsive and -resistant group

Variables	Adjusted OR (95 % CI)	*P* value
Intermediate monocyte	< 0.01 (0.00-0.40)	0.02
Albumin	0.29 (0.05–1.75)	0.18

## Discussions

This study provided immunophenotypes related to the monocyte subtype by IVIG responsiveness for KD. As innate immune cells initiate and propagate the immune responses, monocytes were therefore investigated. We revealed statistically lower proportions of intermediate monocytes in children with IVIG-resistant KD.

The monocyte population is heterogenous that plays an important role as the first line of immune defense and they migrate to inflammatory or infected tissues and can differentiate into macrophages or dendritic cells [10, 11]. Monocytes are recruited by chemokines (such as CD192 or CCDR2) that bind to receptors (CD14, CD16 or CD64) expressed on their cell surface. In human, we have 3 subtypes of monocytes which have different roles in immunity, the classical (CD14 + CD16-), the intermediate (CD14 + CD16+), and the nonclassical (CD14 + CD16++) monocytes [12, 13]. Each subtypes have characteristics unique to their key functions. The classical monocytes have high phagocytic ability, effectively produces inflammatory mediators in response to bacterial products, repairs tissues, and migrates to sites of inflammation. The intermediate monocytes are a newly defined subset which were previously identified within the nonclassical subset. They also produce inflammatory mediators in response to bacteria and has the ability to expand during infection and antigen presentation. The intermediate monocytes have proportionally increased in inflammatory and chronic conditions such as cardiovascular disease, rheumatoid arthritis, and Crohn’s disease, however, the role of intermediate monocytes in such disease has not been investigated [14–18]. Intermediate monocytes actively produce proinflammatory cytokines such as TNF-α, IL-1β and IL-6. Lastly, nonclassical monocytes produce TNF-α, IL-1β and CCL3 in response to viral and immune complex stimulation and they have the ability of FcƔ mediated phagocytosis [19].

Some researchers have demonstrated the role of monocyte/macrophage in KD. In affected tissues in autopsy cases and skin biopsy specimens of KD patients, infiltration of monocytes/macrophages is notable and the number of CD14 + monocytes/macrophages in peripheral blood augmented with increased activation of CD14 + CD23 + monocytes/macrophages during the acute stage of KD [20–22]. In addition, increased peripheral blood CD14 + monocytes/macrophages and secretion of TNF-α, IL-6, and IL-1 were observed more dominantly in KD patients with CAL [9, 22–25]. Koga et al. [7] have demonstrated CD14 + monocytes/macrophages were activated in peripheral blood in the acute stage of KD with exaggerated phagocytosis and strong expression of TNF-α which became weakly expressed in the convalescent stage.

IVIG is a classic treatment modality in KD and those who received IVIG within 10 days of onset have decreased probability of having CAL, but if the patient is resistant to IVIG treatment, the patients have increased probability of developing CAL. The action mechanism of IVIG is not yet fully understood. IVIG plays a role in immune homeostasis by suppressing the activation of innate and adaptive immunity and inhibiting inflammatory mediators to enhance the anti-inflammatory processes [26, 27]. Das et al. have revealed that IVIG induces autophagy in peripheral blood monocytes, monocyte derived from dendritic cells, and M1 macrophages but not in M2 macrophages of healthy donors [28]. Autophagy, a regulated mechanism for clearance of damaged/dysfunctional cells or components to warrant regeneration of new and healthy cells. This process plays a fundamental role in the regulation of innate and adaptive immune responses, lymphocyte differentiation, survival, and homeostasis [29–31]. Furthermore, the concept of autophagy is also important in regulating autoimmune and inflammatory diseases including systemic lupus erythematosus, inflammatory bowel diseases, rheumatoid arthritis, psoriasis, multiple sclerosis and myositis and some report autophagy as a potential target to treat for such diseases [31–35].

Although the mechanism of IVIG action has not been fully elucidated, one proposed mechanism is a blockade of Ig Fc receptors (FcγRs). The bound IgG prevents immune complex from phagocytosis and also from delivering activating signals to the target cells [36]. Abe et al. [36] have reported the correlation of FcγRs expression and KD without sub-dividing them in to IVIG responsive and resistant. In this study, FcγRII expression on monocytes (*n* = 12) was not significantly changed before vs. after IVIG therapy and was not significantly elevated in KD patients compared with febrile controls. FcγRIII expression (*n* = 6) was also down-regulated by IVIG treatment but there was no significant difference in FcγRIII expression levels between pre-IVIG KD patients and controls. The Fc receptor on CD 16 is FcγRIII and this has correlation with intermediate monocyte (CD14 + CD16+) and KD [36].

According to current literatures, approximately 10–20 % of patients are IVIG-resistant, and further research efforts are required to better characterize and predict IVIG-resistant patients [37–40]. Risk factors of IVIG resistance include younger age, male, higher neutrophil count, C-reactive protein (CRP), total bilirubin (TB), aspartate aminotransferase (AST), alanine aminotransferase (ALT) and lactate dehydrogenase (LDH) [2, 37–39, 41]. Scores related to IVIG resistance have been developed using the above variables, but there are discrepancies of performance between Japanese cohorts and North American cohorts [42].

IVIG for acute KD decreases the absolute number of circulating monocytes/macrophages [43]. NF-κB activation in peripheral CD14 + monocytes/macrophages also significantly decreased after IVIG therapy in acute stage of KD, [44] Katayama et al.[8] also observed increased number of CD14 + CD16 + monocytes/macrophages in acute stage of KD which decreased after IVIG infusion.

In this present study, we have evaluated the monocyte subtypes to determine the role of it regarding IVIG responsiveness. The Intermediate monocytes in patients with KD were significantly higher than NFC but significantly lower than OFC. Regarding IVIG responsiveness, the intermediate monocytes were significantly lower in the IVIG-resistant group compared to IVIG-responsive group. This implies that to activate IVIG therapy, a certain number of intermediate monocyte might need initially, however, the decreased numbers and percentage of intermediate monocyte might have weakened the activation of IVIG. Lower level of intermediate monocyte could play a role in IVIG-resistant condition which needs further research whether the intermediate monocyte interfere the action of IVIG. After resolution of fever (after IVIG infusion), the intermediate monocyte decreased significantly in the IVIG-responsive group and while they were increased in the IVIG-resistant group. Decreased percentage of intermediate monocytes in IVIG-responsive group after the resolution of fever was because the proportion of classical monocyte and intermediate monocyte has been changed. While the changes of intermediate monocytes are more noticeable during the acute and subacute phase of KD, the level of intermediate monocytes became similar at convalescent phase of KD. This finding may also imply some interaction between IVIG and the intermediate monocytes and further research is needed.

This study had limitations. First, the small enrollment number of patients with IVIG-resistant, which could limit the statistical analyses. Second, the monocyte profiles between IVIG-responsive and -resistant in KD children were only assessed by the number/proportion of monocyte subtypes. Further functional studies, on the intermediate monocytes in particular, are needed. However, further evaluation into the intermediate monocytes could warrant a step forward to reveal the pathophysiology of KD, especially IVIG-resistant KD.

## Conclusion

In conclusions, the intermediate monocyte may play an important role in IVIG responsiveness. Low starting levels of intermediate monocytes, followed by dramatic increase post-IVIG infusion during acute phase of KD are associated with IVIG-resistance. Functional studies on intermediate monocyte may help to reveal the pathophysiology of KD.

## Data Availability

No.

## Abbreviations

CBC: Complete blood counts
CRP: C-reactive proteins
CAL: Coronary arterial dilatation
ESR: Erythrocyte sedimentation rate
IVIG: Intravenous Immunoglobulin
KD: Kawasaki disease
NLR: Neutrophil to lymphocyte ratio
NFC: Non febrile, healthy controls
OFC: Other febrile controls
WBC: White blood cells
